# Body Burden of Dichlorodiphenyl Dichloroethene (DDE) and Childhood Pulmonary Function

**DOI:** 10.3390/ijerph14111376

**Published:** 2017-11-14

**Authors:** Pallavi P. Balte, Joachim Kühr, Herrman Kruse, Wilfried J. J. Karmaus

**Affiliations:** 1 Division of General Medicine, Columbia University Medical Center, New York, NY 10032, USA; ppb2119@cumc.columbia.edu; 2 Clinic for Pediatric and Adolescent Medicine Klinikum Karlsruhe, Karlsruhe 76133, Germany; Joachim.Kuehr@klinikum-karlsruhe.de; 3 Institute for Toxicology und Pharmacology, University Schleswig-Holstein, Kiel 24105, Germany; kruse@toxi.uni-kiel.de; 4 School of Public Health, Division of Epidemiology, Biostatistics, and Environmental Health, University of Memphis, Memphis, TN 38152, USA

**Keywords:** dichlorodiphenyl dichloroethene, DDE, lung function, FVC, FEV1

## Abstract

Longitudinal studies have shown that early life exposure to dichlorodiphenyl dichloroethene (DDE) can lead to growth reduction during childhood and adolescence. In addition, DDE exposure has been linked to respiratory tract infections and an increased risk of asthma in children. Our aim was to understand the relationships between DDE exposure and pulmonary function in children, and, particularly, whether associations are mediated by the height of the children. We used data from an environmental epidemiologic study conducted in central Germany in children aged 8-10 years. The pulmonary function (forced vital capacity, FVC, and forced expiratory volume in one second, FEV1) were measured in three consecutive years. Blood DDE levels were measured at 8 and 10 years. We used linear mixed models for repeated measurements and path analyses to assess the association between blood levels of DDE and pulmonary function measurements. All models were adjusted for confounders. Linear mixed approaches and modelling concurrent effects showed no significant associations. The path analytical models demonstrated that DDE measured at eight years had significant, inverse, indirect, and total effects on FVC at ten years (*n* = 328; −0.18 L per μg/L of DDE) and FEV1 (*n* = 328; −0.17 L per μg/L of DDE), mediated through effects of DDE on height and weight. The DDE burden reduces pulmonary function through its diminishing effects on height and weight in children. Further studies are required to test these associations in other samples, preferably from a region with ongoing, high DDT application.

## 1. Introduction

Dichlorodiphenyl dichloroethene (DDE) is a principal metabolite of dichlorodiphenyl trichloroethane (DDT), a synthetic chemical belonging to the organochlorine chemicals family, and is recognized as a persistent organic pollutant (POP) [1]. DDT was widely applied as an insecticide until it was banned in most developed countries by the end of 1980s [1,2]. However, it is still largely used in developing countries for malaria control and in agriculture. Because of its lipophilic properties and very long half-life, DDE persists in the environment in all forms of life [1,2]. Humans are exposed to DDE through the food chain, from which it accumulates in adipose tissue, the bloodstream, and the breast [1,2].

Longitudinal studies have shown that both prenatal and postnatal exposure to DDE leads to a reduction in height-related growth and an increase in BMI during childhood [3,4,5]. Not only are the anti-androgenic properties of DDE known to interfere with puberty, reducing the age of menarche [6], but high levels of DDE have also been found in girls with precocious puberty [7]. DDE is also considered to alter both cellular and humoral immunity, with an increased production of cytokines and nitric oxide [8,9,10,11]. Prenatal exposure to DDE has also been linked with respiratory disorders like lower respiratory tract infections, episodes of wheezing, otitis media, and increased risk of asthma in early childhood [12,13,14,15,16].

In children and adolescents, average pulmonary function growth varies greatly according to the height, weight, sex, and age of pubertal growth spurts [17,18,19]. Exposure to DDE may not only disrupt height- and weight-related growth in children, but immunological responses to persistent exposure to DDE may also alter the physiology of airways through remodeling during this critical period of lung growth. Regarding the pulmonary function test (PFT), there are three critical markers: forced vital capacity (FVC), forced expiratory volume in one second (FEV1), and the ratio FEV1/FVC. The latter shows the proportion of the vital capacity that can be expired in the first second of forced expiration, an indicator of airway obstruction. A prior Dutch study demonstrated that perinatal exposure via breastfeeding to dioxins was related to diminished pulmonary function (FEV1/FVC) in a sample of 29 children 7–12 years of age [20]. In 20-year old Danish offspring (*n* = 421), from a cohort of mothers and with a measurement of prenatal exposure to POPs, no association was found between predicted FEV1 and any POPs, including DDE. However, the odds of airway obstruction (defined as FEV1/FVC < 75%) were higher among offspring whose mothers had higher polychlorinated biphenyls, hexachlorobenzene, or DDE serum levels [21]. A Canadian study (*n* = 1696) of adults aged 20–79 years of age demonstrated that lipid-adjusted DDE plasma concentrations were associated with a decline in FVC and FEV1 but not FEV1/FVC [22]. We are not aware of any investigation on DDE exposure and pulmonary function in children.

We hypothesized that DDE exposure affects pulmonary function in childhood, directly or indirectly, via height and weight, in addition to immunological disturbance. To assess these complex relationships, we used a path-analytical approach (structural equation models). Path analysis is an extension of multiple regression, but offers the flexibility of being able to generate estimates for hypothesized causal associations between a set of variables. Path analysis also allows for the inclusion of intervening (mediating) variables in the explanatory models. The model provides direct, indirect, and total effects (their sum) of each variable on the outcome. Their associations can be shown in a causal path diagram [23]. However, to support these assessments, a clear time-order of risk and effects is required.

An environmental epidemiologic study was conducted in central Germany, to investigate the effects of persistent body burden of polychlorinated biphenyls (PCBs) and DDE on health of children [4]. In addition to showing a small reduction in height in girls [4], other results from this study showed that DDE was related to increased total IgE and asthma [14,24]. In this research paper, our aim was to better understand the effects of the DDE body burden on childhood pulmonary function. In particular, we investigated indirect effects of DDE mediated via growth/height. We provide results from repeated measurement analysis followed by results from path analysis.

## 2. Materials and Methods

### 2.1. Study Population

This longitudinal study was conducted in the south of the federal state of Hesse, in central Germany, between 1994 and 1997. Children were recruited from three different regions. Two of these regions located in the Rhine valley were used intensively for vegetable production and were located around industrial waste incinerators, among other industries (e.g., chemical plants); the third region was separated from these regions by small mountains. After obtaining permits from the Data Protection Agency of the State of Hamburg, Germany, from the Ministry of Cultural Affairs of Hesse, Germany, and from the local school committees, parents of 1091 second grade school children in 18 townships were asked to participate in this study. Informed consent, according to the requirements of the Ethical Committee of the Board of Physicians and the Data Protection Agency of the State of Hamburg, was obtained from all participating parents. Parents were asked to let their children participate in phlebotomy only when passive smoking in the private household had not exceeded 10 cigarettes per day in the previous 12 months. We included this restriction to reduce the impact of smoke exposure; however, mothers and fathers sometimes provided different smoking information. Six hundred ninety-one children and their parents participated in the three repeated surveys (December 1994 to April 1995—age 8 years, January to May 1996—age 9 years, January to June 1997—age 10 years). Out of 691 enrolled children, 632 performed pulmonary function tests (PFTs) in the year 1995, 598 in 1996, and 558 in 1997 [4].

### 2.2. Organochlorines in Blood

Blood samples were collected both at baseline and during the last visit two years later. DDE concentrations were determined from 5 mL samples of whole blood by performing high-resolution gas chromatography (HRGC, Model 3400, Varian, Gloucester, Mass) with a 63Ni electron capture detector at the Institute of Toxicology, University of Kiel, Germany. The detection limit was 0.02µg/L for DDE. In addition, reliability was tested with gas chromatography–mass spectometry. The laboratory successfully participated in nationwide interlaboratory quality assessments for DDE and PCB determinations [4].

### 2.3. Pulmonary Function Test

PFTs were conducted at eight (1994–1995), nine (1996), and 10 years (1997) of age using a Masterscope (Software Release 4.0; Erich Jaeger, Würzburg, Germany). The instrument was calibrated daily and each child performed two to three forced expiratory maneuvers, according to the American Thoracic Society (ATS) guidelines, in standing position and wearing a nose clip. Two flow/volume curves were accepted as reproducible if the difference between FVC measurements was ≤5%. The highest FVC and FEV1 values were then selected for statistical analysis. In addition, the ratio FEV1/FVC, indicating the proportion of the vital capacity that can be expired in the first second of forced expiration, was used in some analyses. Before conduction of the PFTs, height and weight were measured each year.

### 2.4. Covariates

Self-administered questionnaires were used in the survey. Information was collected on the child’s age, birth weight, birth order, maternal and paternal education, maternal and paternal height, smoking during pregnancy, and breast-feeding duration. Environmental tobacco smoke (ETS) was assessed as smoking in the child’s home in the previous 12 months (per day: no cigarettes, 1–10 cigarettes, 11–20 cigarettes, 20–30 cigarettes, >30 cigarettes) [4].

### 2.5. Statistical Analysis

For the purpose of this research paper, we used pulmonary function data only from those children who had information on blood levels of DDE either at age 8 years (year 1 of the study) or 10 years (year 3 of the study). DDE exposure was not measured at age 9 years (year 2 of the study), hence for age 9 years we carried forward the DDE values measured at age 8 years. The final data set contained 971 observations with 344 participants. We determined Spearman’s correlations between DDE exposure and height, weight, FEV1, FVC, and FEV1/FVC. We imputed data using multiple imputation methods for missing covariates including history of asthma (*n* = 3), history or maternal (*n* = 5) and paternal asthma (*n* = 10), duration of breastfeeding (*n* = 120), and number of cigarettes smoked during pregnancy (*n* = 7). Next, we used linear mixed models to assess the association between blood levels of DDE and repeated pulmonary function measurements. The compound symmetry covariance structure matrix was selected based on lowest Akaike information criteria and the Bayesian Schwarz information criterion after considering unstructured, compound symmetry, and autoregressive covariance structure matrices. All models were adjusted for age, sex, birth weight, breastfeeding duration, height, weight, smoking during pregnancy, parental history of asthma, and ETS. We applied a backward selection method starting with all potential confounders in the initial model. Then, those confounders remained in the final explanatory model, which changed the pulmonary function estimates for DDE exposure by 10% or more. To account for differential association between height and pulmonary function in boys and girls, we included interaction terms of height with sex.

Finally, we explored the relationship between DDE exposure, height, weight, and pulmonary function in childhood by structural equation analyses (path analyses) using the Covariance Analysis of Linear Structural Equations procedure [25]. Addressing the time-order of “cause” and “effects”, we used path analytical models for longitudinal data with DDE exposure at 8 years of age and lung function at 10 years of age using the Full Information Maximum Likelihood (FIML) method. In addition, to investigate whether associations differ by the sex of the child, we stratified the path analyses by the sex of the child. The adequacy of model fit was determined by several statistics: a chi-square *p*-value > 0.05 indicating data fit if the chi-square test statistic is close to 0, comparative fit index (CFI) > 90, adjusted goodness of fit index (GFI) > 90, and root mean square error of approximation (RMSEA) < 0.06. Statistical analyses were performed using the SAS statistical package (version 9.3; SAS Institute, Cary, NC, USA).

## 3. Results

A total of 328 children (52% of 632 with PFTs) in 1994/95 (age 8 years) and 344 (62% of 558 with PFTs) children in 1997 (age 10 years) had information on DDE. Table 1 shows the demographic, pulmonary function and DDE exposure measures for each year. About 13% had history of asthma. The prevalence of maternal and paternal asthma was about 3% and 5%, respectively. The prevalence of in utero exposure to maternal smoking was 29% (23% + 4% + 1% + 1%), 27%, and 19% at ages 8, 9, and 10 years, respectively. Similarly, the prevalence for ETS at 8, 9, and 10 years was 34%, 31%, and 33% at ages 8, 9, and 10 years, respectively. Although we tried to restrict ETS in the group with DDE measurements not exceeding 10 cigarettes per day, 7–10% of the children at 8, 9, or 10 years of age were exposed to more than 10 cigarettes (Table 1).

Boys had significantly higher FEV1 and FVC than girls in all years, unadjusted for any covariate. However, the FEV1/FVC ratio was higher in girls than in boys, which was due to lower FVC in girls than boys (data not shown). There was weak negative correlation between DDE levels and height, weight, FEV1, and FVC (Table 2).

Results from repeated measures analysis (mixed linear models), assuming direct concurrent effects, showed no significant associations between body burden of DDE and any pulmonary function parameter after adjusting for age, height, weight, sex, breastfeeding status, exposure to in utero maternal smoking, and ETS (Table 3). The interaction of height with sex of the child gained statistical significance for all three outcomes (FVC, FEV1, and their ratio), showing lower FVC and higher FEV1 in girls relative to boys.

**Figure 1:**
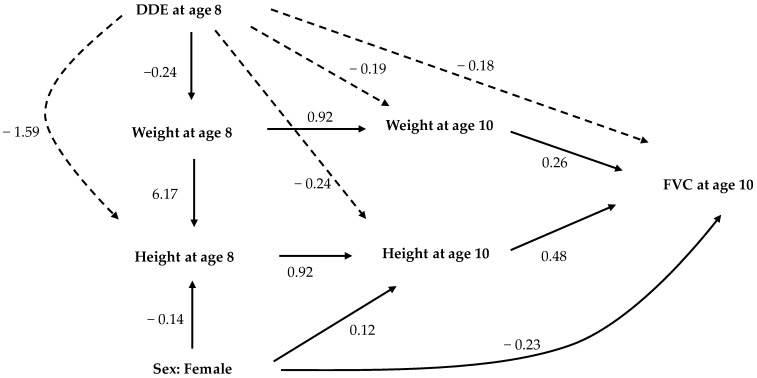
Analytical path model showing statistically significant, standardized, direct, and indirect effects of DDE exposure at eight years of age, height and weight at eight and ten years of age, and FVC at age 10 years. The path coefficients represented by **solid arrows** are direct effects, while those represented by **dashed arrows** are indirect effects. Associations with non-significant total effects are not shown in this diagram. Goodness of fit criteria: χ-squared test statistic = 33.1, *p*-value < 0.05; AGFI = 0.898; CFI = 0.994; RMSEA = 0.048.

**Figure 2:**
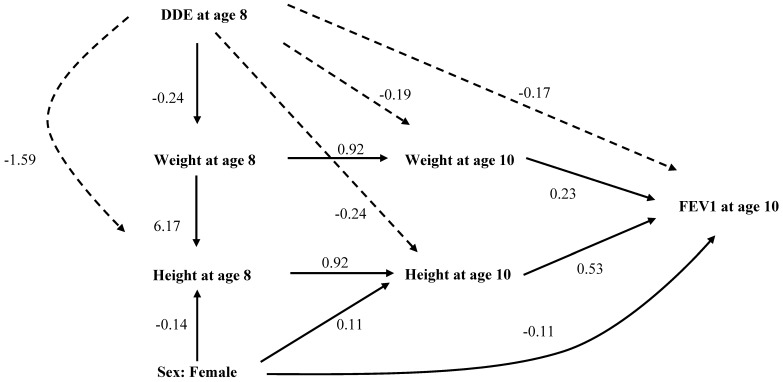
Analytical path model showing statistically significant, standardized, direct, and indirect effects of DDE exposure at eight years of age, height and weight at eight and ten years of age, and FEV1 at age 10 years. The path coefficients represented by **solid arrows** are direct effects, while those represented by **dashed arrows** are indirect effects. Associations with non-significant total effects are not shown in this diagram. Goodness of Fit Criteria: χ-squared test statistic = 33.7, *p*-value < 0.05; AGFI = 0.909; CFI = 0.994; RMSEA = 0.043.

Structural equation models are not restricted to direct effects, but also allow the modeling of indirect effects and the estimation of total effects. To support a “causal” interpretation, we focused on children’s DDE blood concentrations at 8 years of age and their lung functions at 10 years of age (time order of risk and effects). Given this approach, structural equation models with height and weight as intervening variables provided a different picture. Figure 1 and Figure 2 show significant, standardized, direct, and indirect effects of DDE exposure on the height, weight, and sex of the child on FVC and FEV1. The solid arrows indicate direct effects and the dotted arrows the indirect effects. The total effect is the sum of direct and indirect effects. Covariates with non-significant p-value for total effects were not depicted in these path diagrams. The standardized direct, indirect, and total effects of DDE, along with other factors on FVC and FEV1 at age 10 years, are provided in Supplementary Materials (Tables S1–S5).

Results of the structural equation analyses (path analyses) for age 8 years showed that the DDE blood concentration had negative total effects on both height (direct: 1.3 cm per μg/L of DDE, indirect: −0.16 cm per μg/L of DDE, total: −0.28 cm per μg/L of DDE) and weight (total: −0.24 kg per μg/L of DDE) after controlling for effects of age, sex, breastfeeding, and maternal smoking during pregnancy (Table S1). There were significant inverse indirect effects of DDE measured at age 8 years on both height and weight measured at age 10 years, mediated through DDE’s inverse effects on height and weight measured at age 8 years. Similarly, DDE measured at age 8 years had indirect inverse effects on FVC and FEV1 measured at age 10, mediated through its effects on height and weight measured in both years (Figure 1 and Figure 2, Supplemental Tables S3 and S4). We found no direct or indirect effects of DDE on FEV1/FVC (Figure not shown, Supplemental Table S5). In path analyses, female gender was related to lower height both at age 8 and 10 years (Figure 1 and Figure 2). The path coefficients suggested that there was a direct effect of DDE exposure on height, but no direct effect on either FVC or FEV1 (Figure 1 and Figure 2). However, as height and weight explains FVC and FEV1, the diminishing effects of DDE exposure on height and weight were carried forward towards FVC and FEV1 (indirect effect), with height and weight acting as intervening variables (Supplemental Tables S3 and S4).

We also conducted path analysis separately in boys and girls. The stratified analysis had less statistical power to detect differences because of the smaller sample sizes (153 girls and 191 boys). Nonetheless, the path analyses showed that DDE exposure has statisticallly significant, indirect, negative effects on FVC (−0.25 L per μg/L of DDE in females, −0.21 L per μg/L of DDE in males) and FEV1 (−0.24 L per μg/L of DDE in females, −0.19 L per μg/L of DDE in males), both in females and males (Supplemental Figures S1–S4).

## 4. Discussion

We studied the relationships between DDE exposure and pulmonary function in children aged 8 to 10 years in a study conducted in South-Hesse, Germany from 1995–1997. The median DDE levels at baseline (1995) were 0.3 μg/L, which were higher than the median DDE levels of 0.18 μg/L in the age group of 6–10 years measured 15 years later between 2010–2014 [26]. As height and weight are important determinants of pulmonary function, any investigation between exposure of interest and pulmonary function should adjust PFT measurements for height and weight [17,18,19]. However, our exposure of interest, DDE, has been reported to affect height [3,4], and hence adjusting potentially mediating variables such as weight and height will introduce mediator-outcome and/or mediator-exposure confounding [27]. Therefore, we used a novel approach of path analysis, which suggests that DDE blood levels may indirectly reduce FVC and FEV1. DDE exposure has direct inverse effects on reduced height and weight but not on pulmonary function. However, the inverse effect of DDE exposure affected FVC and FEV1, mediated through its effect on height and weight at age 8 and 10 years. Although height and weight continued to have positive direct effects on both FVC and FEV1, these effects were attenuated by the inverse effects of DDE exposure on height and weight.

DDE is known to affect endocrine system through its estrogenic and anti-androgenic properties [2]. Karmaus et al. reported that in utero exposure to DDE is associated with reduced age at menarche by one year in a Michigan angler cohort [6]. High levels of DDE have also been found in girls with precocious puberty who migrated to Belgium from developing countries [28]. Early age at menarche in turn is associated with decreased growth of height post-menarche, thus affecting the final adult height [19,28]. Previous literature on DDE exposure and its effect on height and weight during childhood shows conflicting findings. A prospective study by Ribas-Fito et al. found that increased prenatal DDE concentrations were associated with decreased height at 1, 4, and 7 years of age in both boys and girls [3]. On the other hand, a study by Gladen et al. suggested that prenatal exposure to DDE was associated with increased height at puberty in boys [29]. However, in the same cohort from Germany as used in this study, Karmaus et al. demonstrated that growth during childhood was significantly reduced in girls with high DDE concentrations measured at 8 years of age but no effect was seen in boys [4]. Our path analytical models, used separately in boys and girls, show that DDE has direct negative effects on height and weight, and hence indirect negative effects on FEV1 and FVC (Supplemental Figures S1–S4).

There are some limitations to our study. We did not have DDE exposure measured at age 9 years. Hence, we carried forward the DDE levels measured at age 8 years. However, the median DDE measurements among participants did not differ between ages 8 and 10 years, suggesting a persistent body burden of DDE. Additionally, results from sensitivity analysis using imputed DDE values at 9 years did not differ from the analysis presented in Table 3. This limitation does not affect the path-analytical assessments, since following a clear time-order, only DDE levels at age 8 were used as predictors for FVC at age 10 years.

In addition to its endocrine effects, both animal and human studies have suggested that DDE exposure is also associated with immune dysregulation [9,10,11]. Such dysregulations involving IL-4 can induce epithelial cell proliferation, fibrosis, and mucus secretion in the lungs [9,30]. Thus, DDE-related immune changes may add to the remodeling of both smaller and larger airways, which may explain a higher risk of asthma in children exposed to DDE [13,14,16,31]. Hence, in addition to the mediating effect of height (growth), future investigations should additionally integrate immune markers in the path-analytical models.

One strength of our design is the three-year follow-up that facilitates an assessment of the time order. In addition, DDE exposure, PFT, weight, and height were assessed under standard conditions, reducing information biases. Use of path analysis allowed us to include intervening factors in the explanatory models, which helped disentangle the complex relationship between DDE exposure and pulmonary function. Finally, as in every longitudinal study, missing data was an issue, which we tried to overcome through multiple imputations.

Pulmonary function in children is determined by complex relationships between many factors including age, sex, race, height, weight, and onset of puberty. These relationships are further complicated by exposure to DDE, which is known to affect one or more of these determinants of pulmonary function. Although DDE exposure does not seem to directly influence pulmonary function, it does inversely affect height and weight, which in turn affect pulmonary function. Therefore, poor growth in height and weight at an early age contributes to lower FVC and FEV1. However, whether DDE exposure modifies the physiology of airways leading to lower pulmonary function is unknown. However, our path analyses demonstrated that reductions in pulmonary function parameters are at least partially due to DDE’s effects on height and weight.

Our finding that DDE affects pulmonary function in children is in agreement with two other investigations in adults, one from Denmark and one from Canada [21,22]. Both cohorts adjusted for height. Whereas the Danish sample (age 20 years) with prenatal exposure did not identify associations with FVC and FEV1, it found higher odds of exposure in those with airway obstruction. Regarding FVC and FEV1, it is possible that their negative findings are related to diminished height in more highly exposed children. Hence, adjusting for height will eliminate the effect of DDE, when the exposure occurred early in life. Since the Canadian study focused on adults (20–79 years of age), it is possible that the growth of these adults was not yet effected by exposure when they were children. Consequently, taking height in older adults into account does not lead to over-adjustment in this cohort.

To date, there has been no other study investigating the association between DDE exposure and pulmonary function in children. DDE is a toxic chemical that exists in environment in all forms of life, humans, plants, animals, water, air, and soil, and hence is a major public health concern. Because of its ongoing use for malaria vector control in about 14 countries and additional considerations in other countries to reintroduce DDT [32], future investigations in children that further integrate immune markers into the path-analytical models are necessary.

## 5. Conclusions

In conclusion, the use of structural equation models improved the understanding of underlying relationships between DDE and lung function, allowing the estimation of direct, indirect, and total effects of height, weight, and DDE exposure on pulmonary function. We suggest that exposure to DDE in early childhood may adversely affect pulmonary function and that this effect is partly mediated by height and weight. Hence, when analyzing persistent organic pollutants that may affect the growth of children, we have to refrain from over-simplification, such as adjusting PFTs for height and weight, but we also have to use path-analytical models with height and weight as mediators of DDE. This is a novel finding and certainly warrants further research in other samples of children, preferably from a region with continuous, high DDT application.

## Figures and Tables

**Table 1:** Characteristics of the children with pulmonary function data (years 1995, 1996, and 1997) at ages 8, 9, and 10 years

Characteristic	Age 8 (1995) (*n* = 328)	Age 9 (1996) (*n* = 299)	Age 10 (1997) (*n* = 344)
Age (years), mean (SD)	8.2 (0.5)	9.3 (0.4)	10.3 (0.5)
Female, *n* (%)	143 (44)	126 (42)	153 (45)
Height (cm), mean (SD)	133 (6)	138 (6)	143 (7)
Weight (kg), mean (SD)	30 (6)	33 (7)	39 (9)
FEV1 (L), mean (SD)	1.8 (0.3)	1.9 (0.3)	2.1 (0.3)
FVC (L), mean (SD)	2.1 (0.3)	2.3 (0.3)	2.5 (0.4)
FEV1/FVC (%), mean (SD)	88 (6)	84 (6)	85 (5)
DDE (μg/L), median (IQR)	0.3 (0.2)	0.4 (0.2) *	0.3 (0.2)
Doctor diagnosed asthma, *n* (%)	42 (13)	36 (12)	46 (13)
Maternal asthma, *n* (%)	12 (4)	11 (4)	12 (4)
Paternal asthma, *n* (%)	18 (6)	17 (6)	18 (5)
Breast feeding, *n* (%)	281 (86)	257 (86)	293 (85)
Maternal smoking during pregnancy (per day), *n* (%)			
>30 cigarettes	3 (1)	3 (1)	4 (1)
20–30 cigarettes	3 (1)	3 (1)	4 (1)
11–20 cigarettes	13 (4)	10 (3)	14 (4)
1–-10 cigarettes	74 (23)	66 (22)	78 (13)
Environmental tobacco smoke (per day), *n* (%)			
>30 cigarettes	2 (1)	2 (1)	5 (2)
20–30 cigarettes	5 (2)	4 (1)	5(2)
11–20 cigarettes	19 (6)	16 (5)	21 (6)
1–-10 cigarettes	82 (25)	71 (24)	79 (23)

* DDE values at 9 years of age were carried forward from 8 years of age. The different median resulted from a different number of participants at 8 and 9 years of age.

**Table 2:** Spearman’s correlations for dichlorodiphenyl dichloroethene (DDE) with anthropometric and pulmonary function measures in years 1995, 1996, and 1997.

Variables	Age 8 (1995) (*n* = 328)		Age 9 (1996) (*n* = 299) *		Age 10 (1997) (*n* = 344)	
	Spearman’s correlation	*p*-value	Spearman’s correlation	*p*-value	Spearman’s correlation	*p*-value
DDE (μg/L) - Height (cm)	−0.29	<0.0001	−0.22	0.0001	−0.21	<0.0001
DDE (μg/L) - Weight (kg)	−0.28	<0.0001	−0.23	<0.0001	−0.29	<0.0001
DDE (μg/L) - FEV_1_ (L)	−0.15	0.0107	−0.16	0.0069	−0.15	0.007
DDE (μg/L) - FVC (L)	−0.20	0.001	−0.15	0.0088	−0.18	0.0006
DDE (μg/L) - FEV_1_/FVC (%)	0.08	0.1572	0.01	0.9290	0.09	0.1004

* DDE values at 9 years of age were carried forward from 8 years of age.

**Table 3:** Adjusted linear mixed models for pulmonary function, DDE exposure, height, weight, and sex.

	FVC (L)			FEV1 (L)			FEV1/FVC (%)		
	Estimate	SE	*p*-value	Estimate	SE	*p*-value	Estimate	SE	*p*-value
DDE (μg/L)	0.006	0.02	0.71	0.004	0.02	0.81	0.12	0.43	0.78
Height (cm)	0.03	0.002	<0.0001	0.02	0.002	<0.0001	0.01	0.06	0.84
Weight (kg)	0.01	0.001	<0.0001	0.01	0.002	<0.0001	−0.02	0.04	0.71
Sex: female	−0.48	0.20	0.07	0.14	0.02	0.5	15.8	6.32	0.01
Height×Sex	0.005	0.00	0.007	−0.0004	0.002	0.78	−0.13	0.05	0.004

All models were additionally adjusted for breastfeeding duration, history of maternal smoking during, and current environmental tobacco smoke exposure. Compound symmetry covariance matrix was selected based on lowest Akaike Information Criterion (AIC).

## Supplementary Materials

The following are available online at www.mdpi.com/1660-4601/14/11/1376/s1. Table S1: The standardized direct, indirect, and total effects of DDE and covariates explaining height and weight from path analysis model at 10 years of age, Table S2: The standardized direct, indirect, and total effects of DDE and covariates explaining height and weight based on structural equation models; Table S3: The standardized direct, indirect, and total effects of DDE and covariates explaining FVC (L) at age 10 years using a structural equation model; Table S4: The standardized direct, indirect, and total effects of DDE and covariates explaining FEV1 (L) at age 10 years using a structural equation model; Table S5: The standardized direct, indirect, and total effects of DDE and covariates explaining the ratio FEV1/FVC at age 10 years using a structural equation model; Figure S1: Analytical path model showing statistically significant, standardized, direct, and indirect effects of DDE exposure at eight years, height and weight at eight and ten years, and FVC at age 10 years in boys; Figure S2: Analytical path model showing statistically significant, standardized, direct, and indirect effects of DDE exposure at eight years, height and weight at eight and ten years, and FVC at age 10 years in girls; Figure S3: Analytical path model showing statistically significant, standardized, direct, and indirect effects of DDE exposure at eight years, height and weight at eight and ten years, and FEV1 at age 10 years in boys; Figure S4: Analytical path model showing statistically significant, standardized, direct, and indirect effects of DDE exposure at eight years, height and weight at eight and ten years, and FEV1 at age 10 years.
