# Supplementary material for: Body Burden of Dichlorodiphenyl Dichloroethene (DDE) and Childhood Pulmonary Function
Source: Int J Environ Res Public Health. 2017 Nov 14;14(11):1376. doi: 10.3390/ijerph14111376 (PMC5708015; doi:10.3390/ijerph14111376)
Supplement: Supplementary file 1 [file ijerph-14-01376-s001.pdf]

Table S1. The standardized direct, indirect and total effects of DDE and covariates explaining height and weight from path analysis model at 10 years of age.

Covariates		Height (cm)			Weight (kg)
		**Direct**	**Indirect**	**Total**	**Direct/Total**
DDE at 8 years of age	Estimate	1.3	-1.59	-0.28	-0.24
	SE	0.35	0.38	0.05	0.05
	P-value	0.0002	<0.0001	<0.0001	<0.0001
Age (years)	Estimate	-1.14	1.36	0.2	0.20
	SE	0.33	0.36	0.05	0.05
	P-value	0.0005	0.0002	<0.0001	<0.0001
Weight at 8 years of age	Estimate	6.72		6.72	
	SE	0.23		0.23	
	P-value	<0.0001		<0.0001	
Girls vs. Boys	Estimate	-0.14		-0.14	
	SE	0.04		0.04	
	P-value	0.0003		0.0003	
Being breastfed as child	Estimate	-0.60	0.63	0.03	0.09
	SE	0.41	0.45	0.07	0.07
	P-value	0.1474	0.1650	0.6573	0.1613
Maternal smoking during pregnancy	Estimate	0.05	-0.09	-0.04	-0.01
	SE	0.33	0.36	0.05	0.05
	P-value	0.8684	0.7963	0.4586	0.7963

Goodness of Fit Criteria: Chi-squared test statistic = 5.7, *p*-value < 0.05; AGFI = 0.9991; CFI=0.9936; RMSEA=0.0749. Estimates represent path coefficients with relative importance between -1 and +1. Weight is the first endogenous variable determined by exogenous variables- DDE, age, breastfeeding, and maternal smoking during pregnancy and hence only has direct effects and no indirect effects.

Table S2. The standardized direct, indirect and total effects of DDE and covariates explaining height and weight based on structural equation models. Goodness of Fit Criteria: Chi-squared test statistic = 33.1, *p*-value < 0.05; AGFI = 0.898; CFI = 0.994; RMSEA = 0.048. Estimates represent path coefficients with relative importance between -1 and +1.

**Height at 10 years of age (cm)**									
	**Total**			**Direct**			**Indirect**		
Covariates	Estimate	SE	*p*-value	Estimate	SE	*p*-value	Estimate	SE	*p*-value
DDE at 8 years of age	-0.241	0.05	<0.0001				-0.241	0.05	<0.0001
DDE at 10 years of age	0.018	0.02	0.4313	0.020	0.02	0.3948	-0.001	0.00	0.365
Height at 8 years of age	0.922	0.02	<0.0001	0.922	0.02	<0.0001			
Weight at 8 years of age	1.436	0.54	0.008				1.436	0.54	0.008
Weight at 10 years of age	0.043	0.03	0.1594	0.043	0.03	0.1594			
Age (in years)	0.024	0.02	0.2757	0.023	0.02	0.2961	0.001	0.00	0.3043
Girls vs. Boys	-0.033	0.04	0.3937	0.107	0.02	<0.0001	-0.141	0.04	0.0001
Being breastfed as child	0.043	0.07	0.5174	0.040	0.03	0.1324	0.003	0.06	0.9628
Maternal smoking during pregnancy	0.012	0.05	0.8168	0.026	0.02	0.2229	-0.014	0.05	0.7756
**Weight at 10 years of age (kg)**									
	**Total**			**Direct**			**Indirect**		
Covariates	**Estimate**	**SE**	***p*-value**	**Estimate**	**SE**	***p*-value**	**Estimate**	**SE**	***p*-value**
DDE in year 1995	-0.19	0.05	<0.0001				-0.19	0.05	<0.0001
DDE in year 1997	-0.03	0.02	0.265	-0.03	0.02	0.265			
Weight in year 1995	0.92	0.01	<0.0001	0.92	0.01	<0.0001			
Age (in years)	0.03	0.02	0.16	0.03	0.02	0.16			
Being breastfed as child	0.08	0.07	0.2039	0.03	0.03	0.246	0.05	0.06	0.4054
Maternal smoking during pregnancy	0.01	0.05	0.7914	0.01	0.02	0.6888	0.01	0.05	0.9225

Table S3. The standardized direct, indirect and total effects of DDE and covariates explaining FVC (L) at age 10 years using a structural equation model.

Covariates	Total			Direct			Indirect		
	**Estimate**	**SE**	***p*-value**	**Estimate**	**SE**	***p*-value**	**Estimate**	**SE**	***p*-value**
DDE at 8 years of age	-0.18	0.04	<0.0001				-0.18	0.04	<0.0001
DDE at 10 years of age	0.01	0.04	0.836	0.01	0.03	0.8675	0.002	0.02	0.8945
Age	0.05	0.04	0.1421	0.03	0.03	0.3179	0.02	0.01	0.1759
Height at 8 years of age	0.52	0.04	<0.0001	0.08	0.11	0.4726	0.44	0.10	<0.0001
Weight at 8 years of age	1.04	0.31	0.0007				1.04	0.31	0.0007
Height at 10 years of age	0.48	0.10	<0.0001	0.48	0.10	<0.0001			
Weight at 10 years of age	0.278	0.05	<0.0001	0.26	0.04	<0.0001	0.02	0.02	0.1771
Girls vs. Boys	-0.25	0.04	<0.0001	-0.23	0.04	<0.0001	-0.03	0.02	0.2686
Being breastfed as child	0.0004	0.06	0.9956	-0.04	0.04	0.3166	0.04	0.05	0.397
Maternal smoking during pregnancy	-0.06	0.05	0.252	-0.07	0.04	0.0612	0.01	0.04	0.8344
Environmental tobacco smoke	0.08	0.04	0.0407	0.08	0.04	0.0407			

Goodness of Fit Criteria: Chi-squared test statistic = 33.1, p-value < 0.05; AGFI = 0.898; CFI = 0.994; RMSEA = 0.048. Estimates represent path coefficients with relative importance between -1 and +1.

Table S4. The standardized direct, indirect and total effects of DDE and covariates explaining FEV_1 (L) at age 10 years using a structural equation model.

**Covariates**	**Total**			**Direct**			**Indirect**		
	**Estimate**	**SE**	***p*-value**	**Estimate**	**SE**	***p*-value**	**Estimate**	**SE**	***p*-value**
DDE at 8 years of age	-0.17	0.04	<0.0001				-0.17	0.04	<0.0001
DDE at 10 years of age	0.02	0.04	0.6785	0.02	0.04	0.6909	0.002	0.02	0.9072
Age	0.08	0.04	0.0536	0.06	0.04	0.1345	0.02	0.02	0.1596
Height at 8 years of age	0.51	0.05	<0.0001	0.01	0.12	0.9419	0.51	0.11	<0.0001
Weight at 8 years of age	0.21	0.05	<0.0001				0.21	0.05	<0.0001
Height at 10 years of age	0.53	0.12	<0.0001	0.53	0.12	<0.0001			
Weight at 10 years of age	0.23	0.05	<0.0001	0.23	0.05	<0.0001			
Girls vs. Boys	-0.13	0.04	0.0021	-0.11	0.04	0.0055	-0.02	0.03	0.467
Being breastfed as child	0.04	0.07	0.5065	0.001	0.05	0.9879	0.04	0.05	0.3583
Maternal smoking during pregnancy	-0.09	0.06	0.1203	-0.1	0.04	0.0213	0.01	0.04	0.7723
Environmental tobacco smoke	0.07	0.04	0.1091	0.07	0.04	0.1091			

Goodness of Fit Criteria: Chi-squared test statistic = 33.7, p-value < 0.05; AGFI = 0.909; CFI = 0.994; RMSEA = 0.043. Estimates represent path coefficients with relative importance between -1 and +1.

Table S5. The standardized direct, indirect and total effects of DDE and covariates explaining the ratio FEV_1/FVC at age 10 years using a structural equation model.

**Covariates**	**Total**			**Direct**			**Indirect**		
	**Estimate**	**SE**	***p*-value**	**Estimate**	**SE**	***p*-value**	**Estimate**	**SE**	***p*-value**
DDE at 8 years of age	0.02	0.19	0.9107	-0.01	0.19	0.9574	0.03	0.02	0.0441
DDE at 10 years of age	0.06	0.18	0.7405	0.06	0.18	0.7558	0.004	0.00	0.4165
Age	0.03	0.05	0.6091				-0.001	0.00	0.8015
Height at 8 years of age	-0.06	0.07	0.3977	-0.13	0.17	0.4582	0.07	0.15	0.6749
Weight at 8 years of age	-0.17	0.09	0.0607				-0.17	0.09	0.0607
Height at 10 years of age	0.07	0.17	0.6749	0.07	0.17	0.6749			
Weight at 10 years of age	-0.09	0.07	0.209	-0.09	0.07	0.2035	0.003	0.01	0.6893
Girls vs. Boys	0.3	0.05	<0.0001	0.28	0.05	<0.0001	0.02	0.02	0.4248
Being breastfed as child	0.09	0.07	0.1836	0.09	0.07	0.1684	-0.004	0.01	0.7591
Maternal smoking during pregnancy	-0.06	0.06	0.3292	-0.06	0.06	0.316	0.001	0.01	0.8656
Environmental tobacco smoke	-0.02	0.06	0.765	-0.02	0.06	0.765			

Goodness of Fit Criteria: Chi-squared test statistic = 28.6, p-value < 0.05; AGFI = 0.906; CFI = 0.995; RMSEA = 0.044. Estimates represent path coefficients with relative importance between -1 and +1.

Figure S1. Analytical path model showing statistically significant standardized direct and indirect effects of DDE exposure at eight years, height and weight at eight and ten years, and FVC at age 10 years in boys.

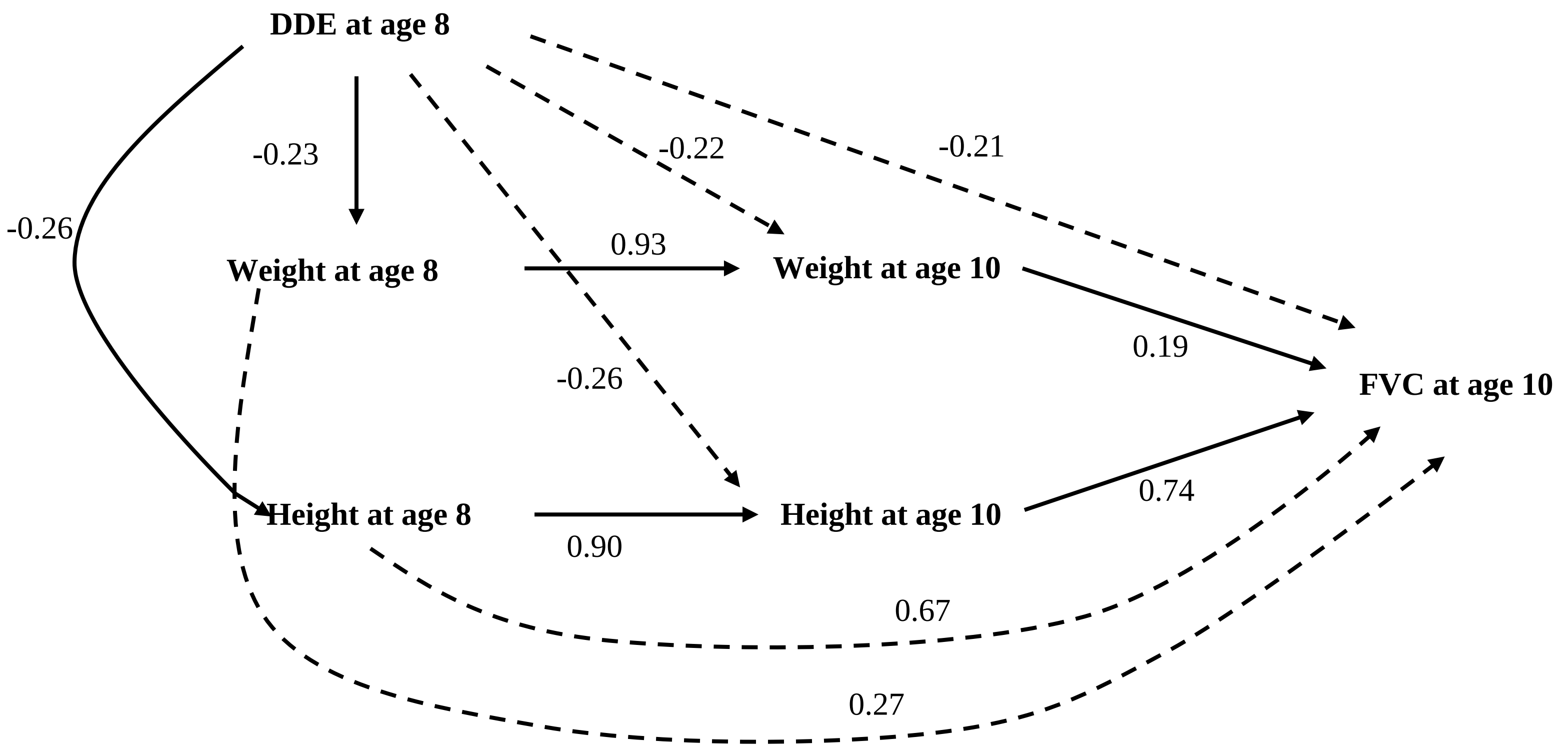

The path coefficients represented by solid arrows are direct effects while those represented by dashed arrows are indirect effects. Associations with non-significant total effects are not shown in this diagram. Goodness of Fit Criteria: Chi-squared test statistic = 31.0, p-value < 0.05; AGFI = 0.899; CFI = 0.985; RMSEA = 0.075.

Figure S2. Analytical path model showing statistically significant standardized direct and indirect effects of DDE exposure at eight years, height and weight at eight and ten years, and FVC at age 10 years in girls.

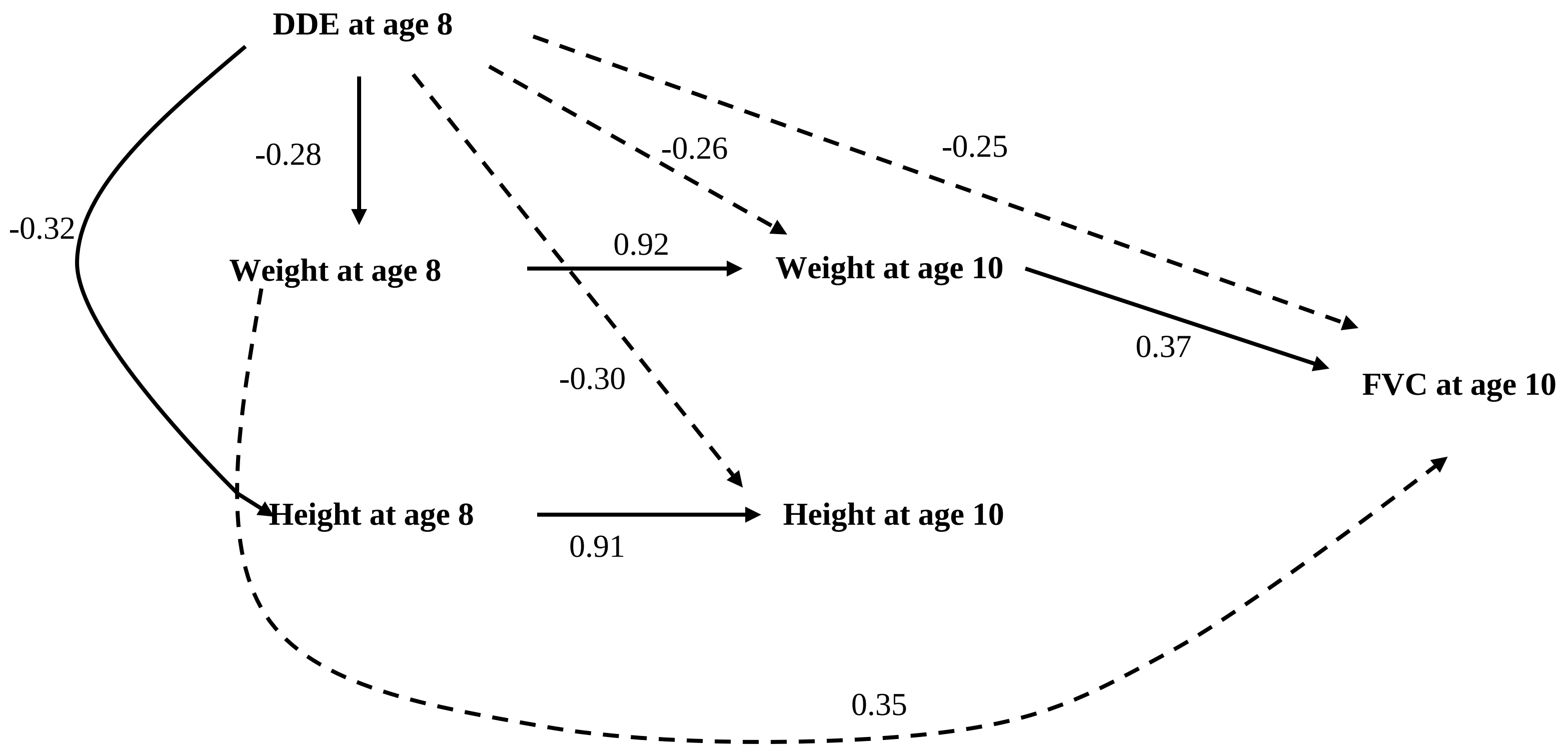

The path coefficients represented by solid arrows are direct effects while those represented by dashed arrows are indirect effects. Associations with non-significant total effects are not shown in this diagram. Goodness of Fit Criteria: Chi-squared test statistic = 18.2, p-value = 0.25; AGFI = 0.89; CFI = 0.996; RMSEA = 0.038.

Figure S3. Analytical path model showing statistically significant standardized direct and indirect effects of DDE exposure at eight years, height and weight at eight and ten years, and FEV_1 at age 10 years in boys.

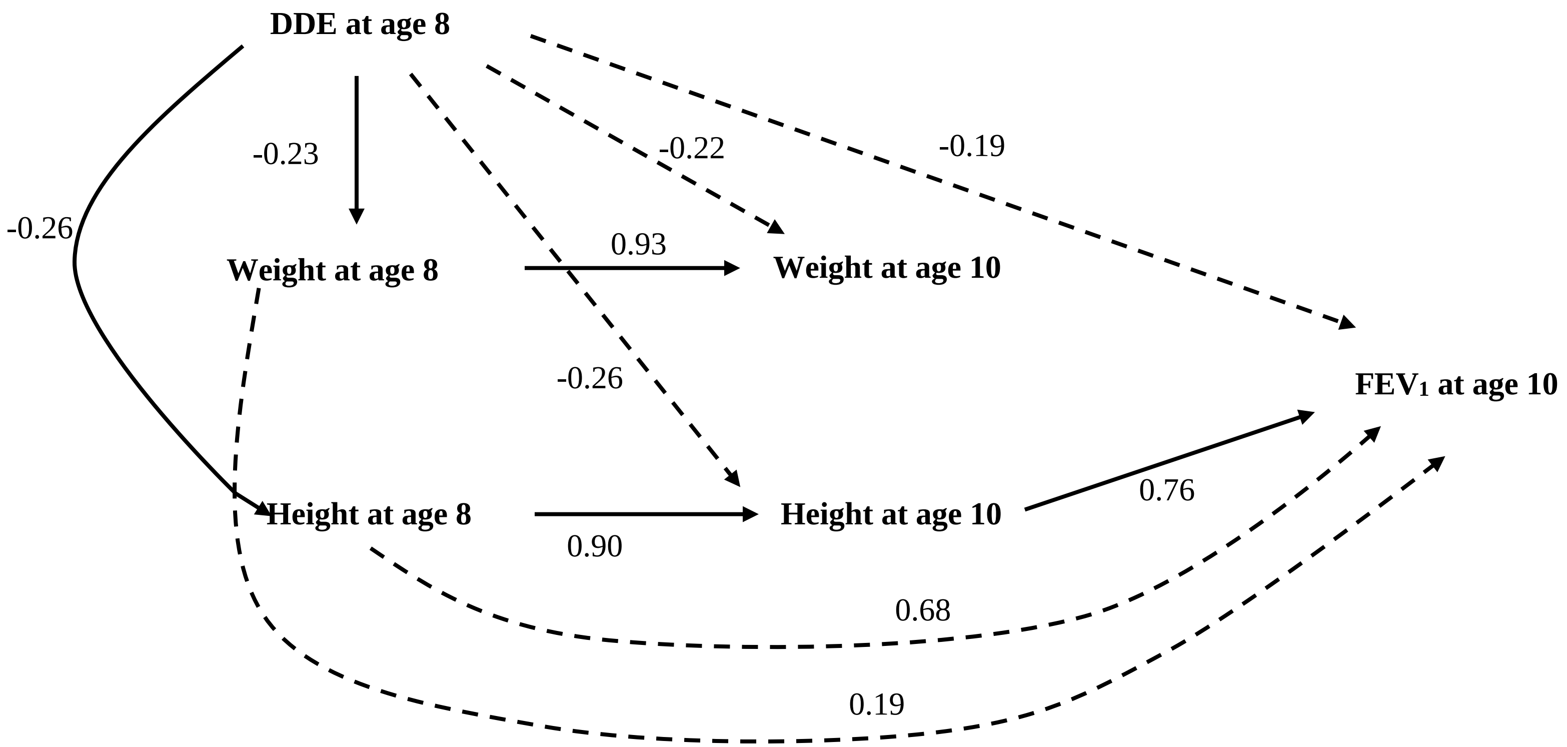

The path coefficients represented by solid arrows are direct effects while those represented by dashed arrows are indirect effects. Associations with non-significant total effects are not shown in this diagram. Goodness of Fit Criteria: Chi-squared test statistic = 32.4, p-value < 0.05; AGFI = 0.863; CFI = 0.983; RMSEA = 0.078.

Figure S4. Analytical path model showing statistically significant standardized direct and indirect effects of DDE exposure at eight years, height and weight at eight and ten years, and FEV_1 at age 10 years in girls.

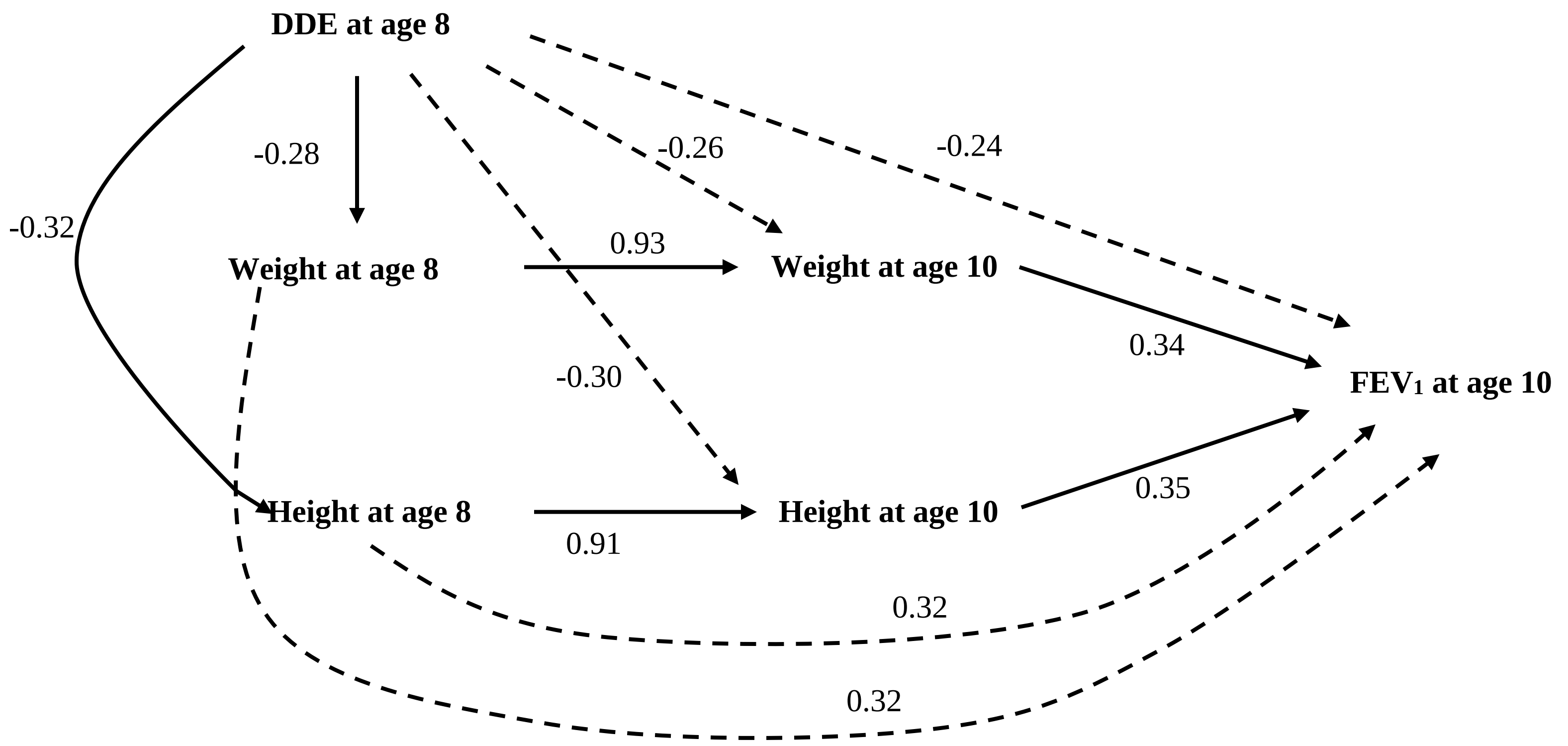

The path coefficients represented by solid arrows are direct effects while those represented by dashed arrows are indirect effects. Associations with non-significant total effects are not shown in this diagram. Goodness of Fit Criteria: Chi-squared test statistic = 17.2, *p*-value = 0.31; AGFI = 0.897; CFI = 0.997; RMSEA = 0.031.
